# Dietary Eriodictyol Alleviates Adiposity, Hepatic Steatosis, Insulin Resistance, and Inflammation in Diet-Induced Obese Mice

**DOI:** 10.3390/ijms20051227

**Published:** 2019-03-11

**Authors:** Eun-Young Kwon, Myung-Sook Choi

**Affiliations:** 1 Department of Food Science and Nutrition, Kyungpook National University, 1370 San-Kyuk Dong Puk-Ku, Daegu 41566, Korea; savage20@naver.com; 2 Center for Food and Nutritional Genomics Research, Kyungpook National University, 1370 San-Kyuk Dong, Puk-Ku, Daegu 41566, Korea

**Keywords:** eriodictyol, insulin resistance, adiposity, hepatic steatosis, inflammation, obesity

## Abstract

The present study aimed to investigate the molecular mechanisms underlying the anti-obesity effect of flavonoid eriodictyol (ED) supplementation in mice fed with a high-fat diet (HFD). C57BL/6N mice were fed with normal diet (ND), HFD (40 kcal% fat), or HFD + 0.005% (*w*/*w*) ED for 16 weeks. In HFD-induced obese mice, dietary ED supplementation significantly alleviated dyslipidemia and adiposity by downregulating the expression of lipogenesis-related genes in white adipose tissue (WAT), while enhancing fecal lipid excretion. ED additionally improved hepatic steatosis and decreased the production of pro-inflammatory cytokines by downregulating the expression of hepatic enzymes and the genes involved in lipogenesis and upregulating the expression of hepatic fatty acid oxidation-related enzymes and genes. In addition, ED improved insulin resistance (IR) by suppressing hepatic gluconeogenesis, enhancing glucose utilization, and modulating the production and release of two incretin hormones, namely gastric inhibitory polypeptide (GIP) and glucagon-like peptide-1 (GLP-1). Taken together, the current findings indicated that ED can protect against diet-induced obesity and related metabolic disturbances, including dyslipidemia, inflammation, fatty liver disease, and IR in diet-induced obese mice.

## 1. Introduction

High-fat food products are highly consumed, particularly in the United States, leading to high rates of obesity, which represents one of the most severe burdens on healthcare systems. Obesity has been closely linked to metabolic syndrome and is often accompanied by increased adiposity, dyslipidemia, chronic inflammation, insulin resistance (IR), non-alcoholic fatty liver disease (NAFLD), and non-alcoholic steatohepatitis (NASH) [1]. Although the mechanisms underlying abdominal obesity and metabolic syndrome are not fully understood, the dysregulation of lipid metabolism in liver and white adipose tissue (WAT) is considered a significant factor that contributes to adiposity and obesity-related complications [2].

Eriodictyol (ED) is a tetrahydroxyflavanone, a flavanone compound substituted by hydroxy groups at positions 5, 7, 3′, and 4′. ED is abundantly produced by numerous medicinal plants, such as *Bauhinia ungulata* [3], *Arcytophyllum thymifolium* [4], *Elsholtzia bodinieri* [5], and *Clinopodium chinense* [6]. ED exhibits anti-inflammatory and anti-oxidative activities and thus exerts protective effects on the neurons, kidneys, and lungs [7]. Chronic inflammation is common in obese individuals and it is strongly linked to insulin resistance [8]. These observations suggest that ED is probably effective in preventing obesity and its complications. However, no evidence associated with the anti-obesity action of ED has been reported so far, and the involved mechanisms are still not clear. Therefore, the present study was designed to elucidate the metabolic effects of ED in diet-induced obese (DIO) C57BL/6N mice. The present study is the first to demonstrate that dietary ED attenuates obesity-mediated metabolic disorders, including adiposity, dyslipidemia, inflammation, NAFLD, and IR in DIO mice.

## 2. Results

### 2.1. Eriodictyol Supplementation Reduced Body Fat and Deposition and Regulated Gene Expression in Adipocytes

**Figure 1:**
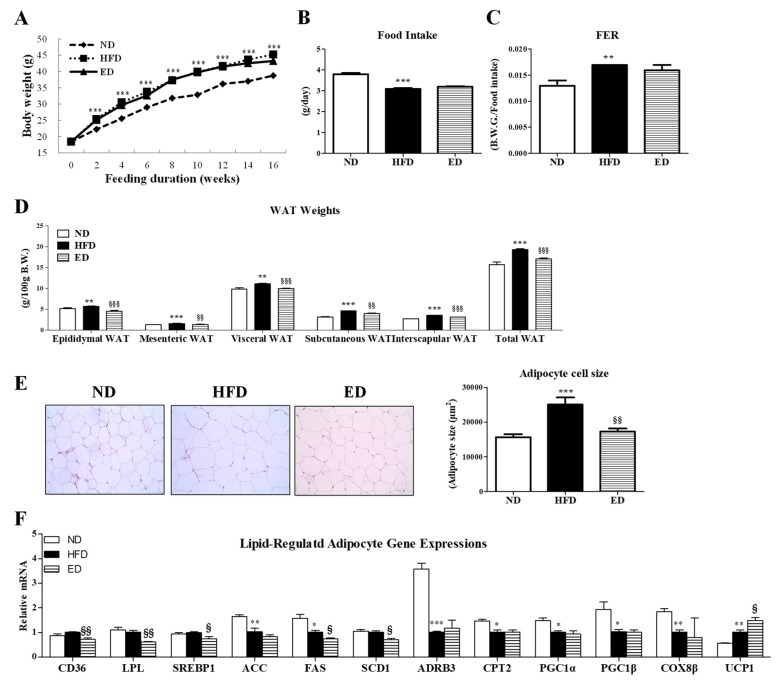
Effect of eriodictyol treatment on body weight (BW) (**A**), food intake (**B**), food efficiency ratio (**C**), white adipose tissue (WAT) weights (**D**), WAT morphology (200× magnification) and adipocyte cell size (**E**), and expression of adipocyte genes (**F**) in C57BL/6N mice fed with the high-fat diet (HFD). Data are presented as means ± SEM. Normal diet (ND; AIN-76) vs. HFD; ^*^ *p* < 0.05, ^**^ *p* < 0.01, ^***^ *p* < 0.001. HFD vs. ED (HFD + 0.005% ED); ^§^ *p* < 0.05, ^§§^ *p* < 0.01, ^§§§^ *p* < 0.001. Cd36, cd antigen 36; LPL, lipoprotein lipoase; SREBP1, sterol regulatory element-binding transcription factor 1; ACC, acetyl-CoA carboxylase; FAS, fatty acid synthase; SCD1, steroly-CoA desaturase 1; ADRB3, adrenoreceptor β3; CPT2, carnitine palmitoyltransferase 2; PGC1, peroxisome proliperator-acivated receptor γ coactivator 1; COX8β, cytochrome c oxidase subunit 8β; UCP1, uncoupling protein 1.

Eriodictyol (ED) supplementation did not alter the body weight, food intake, and food efficiency ratio (FER) in high-fat diet (HFD)-induced obese mice (Figure 1A–C). However, mice fed with the ED-supplemented diet showed significant reduction in the weight of epididymal, mesenteric, visceral, subcutaneous, interscapular, and total white adipose tissue (WAT), as well as reduced epididymal adipocyte size (Figure 1D,E). Moreover, relative to mice in the HFD group, mice in the ED group showed downregulated expression of adipocyte genes involved in lipid uptake (i.e., *CD36*, and *LPL*) and lipogenesis (*SREBP1*, *ACC*, and *SCD1*) accompanied by upregulated expression of the *UCP1* gene, which is involved in FA oxidation (Figure 1F). However, mice in the HFD and the ED groups showed no significant changes in the expression of *ADRB3*, *CPT2*, *PGC1α*, *PGC1β*, and *COX8β* genes, which are involved in fatty acid oxidation (Figure 1F).

### 2.2. Eriodictyol Supplement Improved Plasma and Hepatic Lipid Levels, and Modulated the Expression of Hepatic Lipid-Regulating Enzymes and Genes

**Figure 2:**
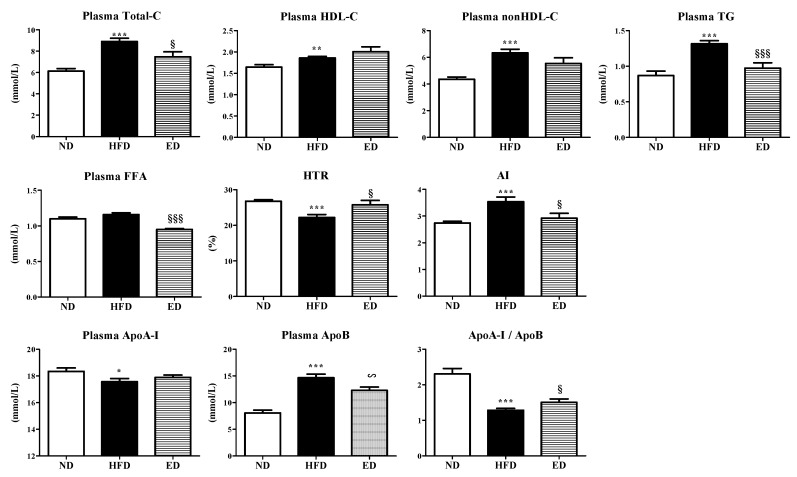
Effect of eriodictyol treatment on the plasma lipid levels in C57BL/6N mice fed with the high-fat diet (HFD). Data are presented as means ± SEM. Normal diet (ND; AIN-76) vs. HFD; ^*^ *p* < 0.05, ^**^ *p* <0.01, ^***^ *p* < 0.001. HFD vs. ED (HFD + 0.005% ED); ^§^ *p* < 0.05, ^§§§^ *p* < 0.001. Total-C, total-cholesterol; HDL-C, high-density lipoprotein-cholesterol; TG, triglyceride; FFA, free fatty acid; HTR, HDL-cholesterol-to-TC ratio; AI, atherogenic index; Apo, apolipoprotein.

The results revealed that ED supplementation in HFD-fed mice significantly reduced plasma total-cholesterol (TC), triglyceride (TG), and free fatty acid (FFA) levels, as well as atherogenic index (AI) and apolipoprotein B (ApoB) levels. In addition, ED-supplemented HFD-fed mice showed markedly higher HDL-cholesterol-to-TC ratio (HTR) and ApoA-I/ApoB ratio (Figure 2).

**Figure 3:**
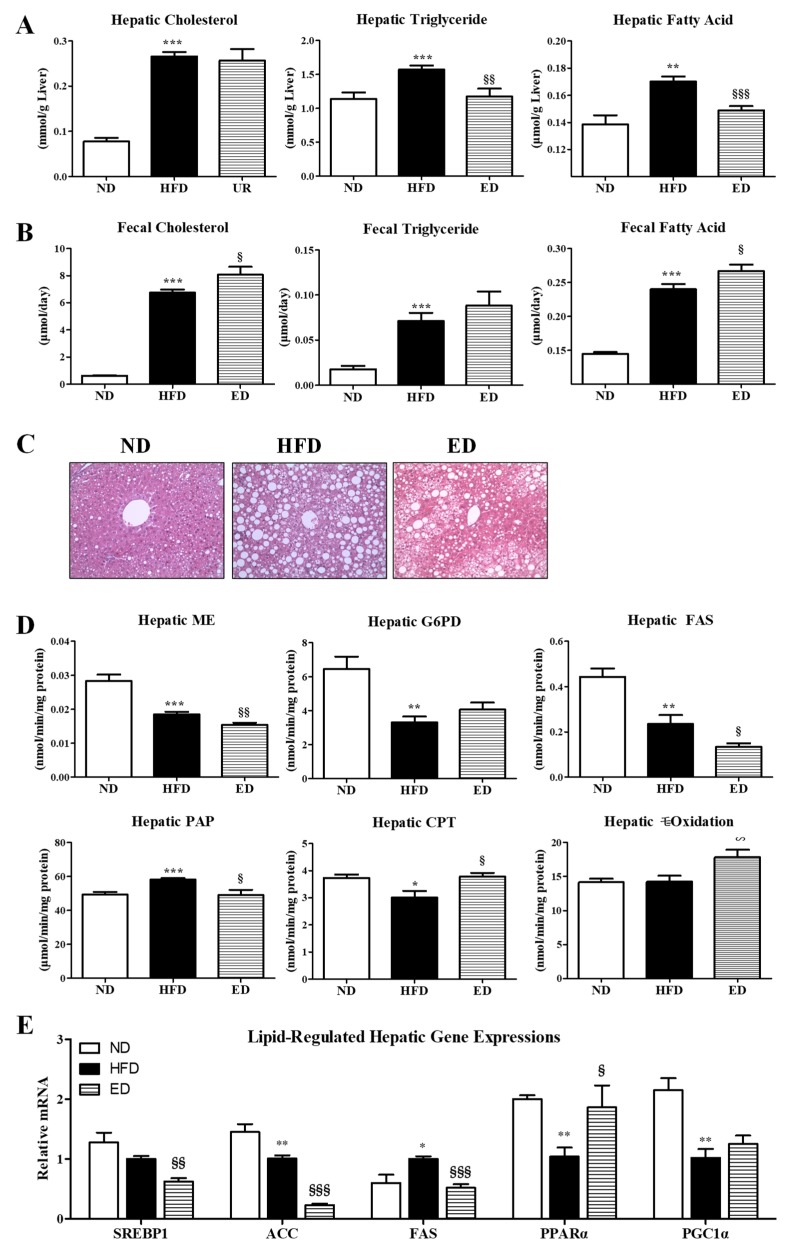
Effect of eriodictyol treatment on hepatic lipid levels (**A**), fecal lipid levels (**B**), hepatic morphology (200× magnification) (**C**), activities of hepatic lipid-regulating enzymes (**D**), and expression of hepatic genes (**E**) in C57BL/6N mice fed with the high-fat diet (HFD). Data are presented as means ± SEM. Normal diet (ND; AIN-76) vs. HFD; ^*^ *p* < 0.05, ^**^ *p* <0.01, ^***^ *p* < 0.001. HFD vs. ED (HFD + 0.005% ED); ^§^ *p* < 0.05, ^§§^ *p* < 0.01, ^§§§^ *p* < 0.001. ME, malic enzyme; G6PD, glucose-6-phosphate dehydrogenase; FAS, fatty acid synthase; PAP, phosphatidate phosphohydrolase; CPT, carnitine palmitoyltransferase; SREBP1, sterol regulatory element-binding transcription factor 1; ACC, acetyl-CoA carboxylase; PPARα, peroxisome proliferator-activated receptor α; PGC1α, PPAR-γ coactivator 1 α.

Moreover, mice in the ED group showed dramatically reduced hepatic TG and FA levels, relative to those in the HFD group, accompanied by markedly increased fecal excretion of cholesterol and FA (Figure 3A,B). The results of hematoxylin and eosin (H&E) staining revealed that the sizes and numbers of hepatic lipid droplets were decreased in the ED group compared to those in the HFD group (Figure 3C). ED significantly decreased the enzymatic activity of malic enzyme (ME), fatty acid synthase (FAS), and phosphatidate phosphohydrolase (PAP), and downregulated the gene expression of *SREBP1*, *ACC*, and *FAS*, which are known to regulate lipogenesis, in the livers of HFD-fed mice. In addition, ED markedly upregulated the expression of fatty acid oxidation-associated enzymes (carnitine palmitoyltransferase (CPT) and β-oxidation) and the *PPARα* gene in the livers of HFD-fed mice (Figure 3D,E).

### 2.3. Eriodictyol Supplementation Ameliorated Insulin Resistance, Glucose Tolerance, and Adipokine/Cytokine Dysregulation

**Figure 4:**
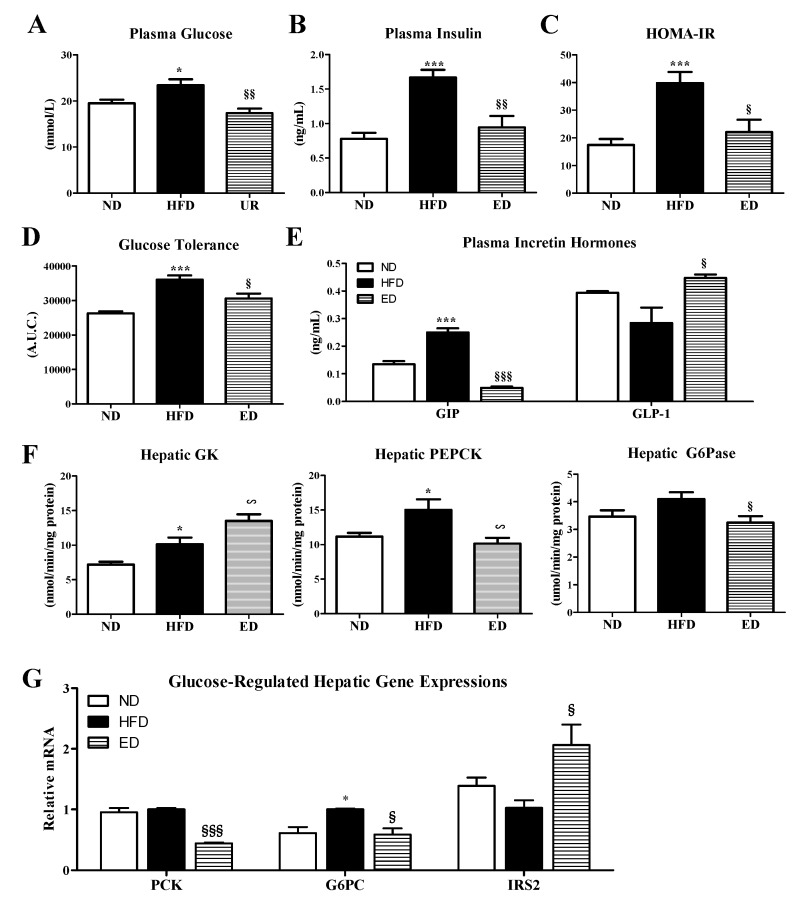
Effect of eriodictyol treatment on the plasma glucose (**A**) and insulin (**B**) levels, HOMA-IR (**C**), glucose tolerance (**D**), plasma incretin hormones (**E**), activities of hepatic glucose-regulating enzyme (**F**), and expression levels of hepatic glucose-regulating genes in C57BL/6N mice fed with the high-fat diet (HFD). Data are presented as means ± SEM. Normal diet (ND; AIN-76) vs. HFD; ^*^ *p* < 0.05, ^***^ *p* < 0.001. HFD vs. ED (HFD + 0.005% ED); ^§^ *p* < 0.05, ^§§^ *p* < 0.01, ^§§§^ *p* < 0.001. HOMA-IR, homeostasis model assessment of insulin resistance; GIP, gastric inhibitory polypeptide; GLP-1, glucagon-like peptide-1; GK, glucokinase; PEPCK, phosphoenolpyruvate carboxykinase; G6Pase, glucose-6-phosphatase; PCK, PEPCK; G6PC, G6Pase; IRS2, insulin receptor substrate 2.

ED-supplemented mice showed significantly reduced plasma glucose and insulin levels relative to mice in the HFD group (Figure 4A,B). Furthermore, ED-supplemented HFD mice showed improved insulin resistance based on the results of an intraperitoneal glucose tolerance test (IPGTT) and a homeostasis model assessment of insulin resistance (HOMA-IR) index relative to mice in the HFD group (Figure 4C,D). Moreover, the ED supplementation significantly decreased the circulating levels of the gastric inhibitory polypeptide (GIP), which stimulates insulin secretion; however, ED supplementation increased the plasma levels of glucagon-like peptide-1 (GLP-1), which increases insulin sensitivity and activity (Figure 4E). In addition, the expression of hepatic phosphoenolpyruvate carboxykinase (PEPCK) and glucose-6-phosphatase (G6Pase) enzymes and their encoding genes (*PCK* and *G6PC*) were significantly down-regulated, whereas hepatic glucokinase (GK) activity and insulin receptor substrate 2 (*IRS2*) gene expression level was dramatically increased in the ED-supplemented group compared to those in the HFD group (Figure 4F,G).

**Figure 5:**
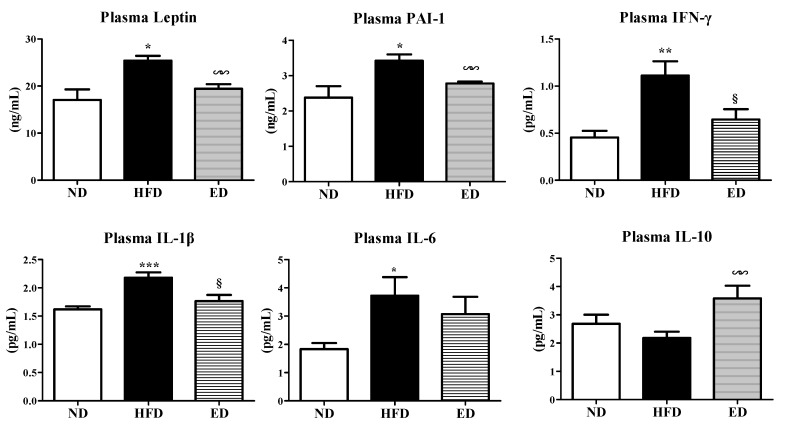
Effect of eriodictyol treatment on the plasma adipokine and cytokine levels in C57BL/6N mice fed with the high-fat diet (HFD). Data are presented as means ± SEM. Normal diet (ND; AIN-76) vs. HFD; ^*^ *p* < 0.05, ^**^ *p* <0.01, ^***^ *p* < 0.001. HFD vs. ED (HFD + 0.005% ED); ^§^ *p* < 0.05, ^§§^ *p* < 0.01. PAI-1, plasminogen activator inhibitor-1; IFN-γ, interferon-gamma; IL, interleukin.

ED supplementation significantly reduced the plasma levels of pro-inflammatory cytokines, including plasminogen activator inhibitor-1 (PAI-1), interferon γ (IFN-γ), and interleukin 1 β (IL-1β), as well as adipokine leptin. Furthermore, ED supplementation increased the plasma levels of IL-10, a cytokine with potent anti-inflammatory properties (Figure 5).

## 3. Discussion

HFD is known to cause obesity, which is linked with a higher risk of metabolic complications, such as dyslipidemia, adiposity, hepatic steatosis, chronic inflammation, and IR, through multiple mechanisms. In the present study, long-term ED supplementation significantly attenuated the increase in HFD-induced WAT weight without altering the food intake and body weight in HFD-fed mice. Similar results were reported in a previous study [9], in which mice fed with a high-fat and high-sucrose (HF/HS) with ED diet for 8 weeks showed no significant differences in body weight but showed a moderate reduction in the weights of perirenal and epididymal fat relative to the HF/HS group. Furthermore, ED supplementation significantly downregulated the expression of the adipocyte genes involved in FA uptake (*CD36* and *LPL*) and lipogenesis (*SREBP1*, *FAS*, and *SCD*), which could be associated with reduced adiposity observed in ED-fed obese mice.

Multiple epidemiologic studies have investigated the effects of HFD on NAFLD and obesity [10,11]. Furthermore, the metabolic profiles of the majority of patients with obesity and metabolic syndrome (MS), which includes NAFLD and dyslipidemia, were found to be characterized by high TC, LDL-C, TG, and FFA, and low HDL-C levels [12]. In this respect, dyslipidemia is closely linked to obesity, NAFLD, and MS. NAFLD is characterized by increased circulating concentrations of FFAs, excess lipid accumulation in the liver, hepatocyte apoptosis, and progressive inflammation. Inflammation significantly contributes to fibrogenesis, leading to NASH, a severe form of NAFLD. In the present study, ED supplementation was found to reduce the plasma levels of TC, TG, and FFA and decrease the atherogenic index, indicating improved dyslipidemia. Notably, ED supplementation significantly increased fecal excretion of cholesterol and FA, which appeared to promote the beneficial effects of ED on dyslipidemia by suppressing the absorption of exogenous fats. In addition to improving dyslipidemia, ED markedly reduced hepatic lipid droplet accumulation and lipid content (TG and FA). Higher FFA plasma levels were found to induce FFA accumulation in hepatocytes by increasing hepatic fatty acid uptake, which interferes with lipid metabolism and enhances lipogenesis, thereby leading to the progression of NAFLD and lipotoxic hepatocellular injury [13]. In the present study, ED not only significantly reduced hepatic lipogenesis by decreasing the activities of ME, FAS, and PAP and downregulating the expression of the *SREBP1*, *ACC*, and *FAS* genes, but also markedly increased the expression of enzymes (CPT and β-oxidation) and the *PPARα* gene, which regulates fatty acid oxidation, in the liver of HFD-fed mice. The above findings suggested that the coordinated action of increased fecal excretion of FA and cholesterol, attenuated dyslipidemia, decreased hepatic lipogenesis, and enhanced hepatic fatty acid oxidation by ED supplementation appeared to attenuate hepatic steatosis. Furthermore, ED decreased the levels of plasma pro-inflammatory cytokines, including PAI-1, IFN-γ, and IL-1β, and increased the anti-inflammatory cytokine IL-10 level in the plasma, suggesting that ED exerts a protective role against the development of NASH/NAFLD.

NAFLD with adiposity and dyslipidemia are strongly associated with IR [14]. Moreover, IR is one of the multiple factors that influences disease progression from NAFLD to NASH [15]. In the present study, we also observed that ED decreased the plasma glucose and insulin levels, thereby improving IR based on IPGTT and the reduced HOMA-IR measurements. In addition, ED-supplemented HFD mice showed dramatically reduced circulating GIP levels and higher plasma levels of GLP-1 relative to the HFD mice. Between the two incretin hormones GIP and GLP-1, GIP is involved in lipid metabolism, and elevated plasma GIP level promotes hyperinsulinemia and obesity-induced IR development [16,17]. By contrast, GLP-1 exerts anti-inflammatory effects against NAFLD, and the increase in GLP-1 secretion ameliorates IR in HFD-induced obese mice [16]. In the fasting state, glucose production via gluconeogenesis is primarily regulated by the liver, and fasting hyperglycemia occurs as a result of dysregulated expression of hepatic gluconeogenic enzymes and genes, such as PEPCK and G6Pase [18]. Thus, the current findings indicated that the suppression of gluconeogenesis via downregulation of the expression of hepatic PEPCK and G6Pase enzymes and their genes is associated with improved insulin sensitivity and activity in ED-supplemented obese mice. 

Moreover, ED supplementation significantly enhanced glucose utilization by increasing the activity of hepatic GK, which is involved in glycolysis, and by upregulating hepatic IRS2 mRNA expression. A previous study using IRS2^−/−^ mice suggested that IRS2-deficient mice developed diabetes as a result of severe IR paired with β-cell failure [19] and showed higher lipogenic enzyme FAS and hepatic lipid levels [20]. Therefore, the above findings suggested that attenuation of NAFLD by ED supplementation is partially linked with glucose homeostasis and leads to the prevention of insulin insensitivity and IR.

Adiposity is linked with the production and release of several inflammatory mediators, which can cause chronic low-grade inflammation and IR [21,22]. Leptin is an adipokine that is primarily expressed in adipose tissue and plays a major role in body weight regulation. Leptin can modulate the production of pro-inflammatory adipokines/cytokines [23]. Obesity and its associated complications, such as IR, are associated with increased plasma leptin concentrations [24,25,26]. The above findings indicated that leptin acts as a potential link between inflammation and IR. ED treatment significantly lowered plasma levels of leptin and markedly decreased the levels of plasma pro-inflammatory cytokines, thereby improving inflammation coupled with adiposity, which in turn prevents IR.

## 4. Materials and Methods

### 4.1. Experimental Animals and Diet

C57BL/6N mice (4 weeks old, male) were obtained from the Jackson Laboratory (Bar Harbor, ME, USA). All mice were individually housed under controlled temperature conditions (24 °C) with the 12 h light-dark cycle and fed with a normal chow diet for 1 week of acclimation. The mice were then randomly assigned (*n* = 10 mice/group) to one of the following experimental diets for 16 weeks: ND (AIN-76 purified diet), HFD (20% fat based on AIN-76 diet plus 1% cholesterol), or HFD with 0.005% (*w*/*w*) of ED. The HFD contained 40 kcal% fat, 17 kcal% protein and 43 kcal% carbohydrate with the fat sources consisting of lard (85% (*w*/*w*) of total fat) and corn oil (15% (*w*/*w*) of total fat). At the end of the experimental period, the mice were anesthetized with isoflurane (5 mg/kg body weight; Baxter, Deerfield, IL, USA) after 12 h of fasting. After blood collection to determine the plasma lipid, adipokine, and hormone concentrations, liver and WAT tissues were removed, and samples were rinsed with physiological saline, weighed, snap-frozen in liquid nitrogen, and stored at −70 °C until enzymatic activity determination and/or RNA analysis. The animal study protocols were approved by Kyungpook National University Ethics Committee (Approval No. KNU 2016-0040, April 18, 2016).

### 4.2. Blood Analysis

The plasma TC, HDL-C, TG, and glucose concentrations were determined using commercial kits (Asan Pharm Co., Seoul, South Korea). The plasma FFA concentration was determined using a commercial kit (Wako Chemicals, Osaka, Japan). The ApoA-I and ApoB100 levels were additionally measured using enzymatic kits (Eiken Chemical Co., Tokyo, Japan). The non HDL-C, HTR, and AI values were calculated as follows: non HDL-C = (TC) − (HDL-C); HTR (%) = (HDL-C) / (TC) × 100; and AI = [(TC) − (HDL-C)] / (HDL-C). The plasma insulin, GIP, GLP-1, leptin, PAI-1, IFN-γ, IL-1β, IL-6, and IL-10 levels were measured using a MILLIPLEX kit (Merck Millipore, Billerica, MA, USA). HOMA-IR was calculated as (fasting glucose (mmol/L) × fasting insulin (μIU/mL))/22.5. For the glucose tolerance test, mice were fasted for 12 h at 15 weeks after the start of the diet experiments and then injected intraperitoneally with glucose (0.5 g/kg body weight). Blood glucose levels were measured from the tail vein with a glucose analyzer (One Touch Ultra, Wayne, PA, USA) at 0, 30, 60, and 120 min after glucose injection.

### 4.3. Hepatic and Fecal Lipid Contents

Hepatic and fecal lipids were extracted following the method of Folch et al. [27]. Fecal samples from each group were collected daily for 1 week and subjected to lipid extraction. The dried hepatic lipid residues were dissolved in 1 mL of ethanol for TG, cholesterol, and FA assays. Triton X-100 and a sodium cholate solution in distilled water were added to 200 μL of the dissolved lipid solution for emulsification. The fecal samples were dried and extracted in ice-cold chloroform and methanol (2:1, *v/v*) for 24 h at 4 °C. After centrifugation at 900 g for 10 min, the supernatant was collected, dried at 50 °C and dissolved in ethanol. Hepatic and fecal TG, cholesterol, and FA concentrations were analyzed using the same enzymatic kits used for the plasma analyses.

### 4.4. Activities of Hepatic Lipid- and Glucose-Regulating Enzymes

Hepatic mitochondrial, cytosolic, and microsomal fractions were prepared as previously described [28] with slight modifications. Protein concentrations were determined by the Bradford method [29]. Cytosolic FAS activity was measured by monitoring the malonyl coenzyme A-dependent oxidation of NADPH at 340 nm [30]. Cytosolic ME activity was measured according to the method described by Ochoa et al. [31] by monitoring the cytosolic NADPH production at 340 nm. The G6PD activity was determined in the cytosol following the method of Pitkanen et al. [32] based on the reduction of 1 μmol of NADP per minute at 25 °C measured at 340 nm using a spectrophotometer. Mitochondrial fatty acid β-oxidation was measured by monitoring the reduction of NAD+ to NADH at 340 nm [33]. Mitochondrial CPT activity was assayed using the method described by Markwell et al. [34]. Microsomal PAP activity was determined following a spectrophotometric method [35]. GK activity in the cytosol was measured using a spectrophotometric assay as described by Davidson and Arion [36], in which the formation of glucose-6-phosphate at 37 °C was coupled to its oxidation by glucose-6-phosphate dehydrogenase and NAD+. PEPCK activity was measured relative to the rate of oxaloacetate synthesis and the reduction of NADH to NAD following the spectrophotometric assay developed by Bentle and Lardy [37]. G6Pase activity in the microsomes was determined following the method described by Alegre et al. [38] with slight modifications.

### 4.5. RNA Isolation and Gene Expression Analysis

The liver and epididymal WAT were homogenized in TRIzol reagent (Invitrogen, Grand Island, NY, USA). Total RNA was isolated according to the manufacturer’s instructions. DNase digestion was performed to remove contaminating DNA. RNA purity and integrity were evaluated using the Agilent 2100 Bioanalyzer (Agilent Technologies, Palo Alto, CA, USA). Total RNA (1 μg) was reverse-transcribed into cDNA using the QuantiTect reverse transcription kit (Qiagen, Hilden, Germany). mRNA expression levels were quantified by real-time RT-PCR using the SYBR green PCR kit (Qiagen) and the CFX96TM real-time system (Bio-Rad, Hercules, CA, USA). The gene-specific primers used for real-time RT-PCR are presented in Table 1. The following amplification profile was used: 35 cycles of 10 min at 90 °C, 15 s at 95 °C, and 60 s at 60 °C. Cycle thresholds were determined based on SYBR green emission intensities during the exponential phase. The Ct data were normalized using *GAPDH*, and the relative gene expression levels were calculated using the 2^∆∆*C*t^ method.

### 4.6. Morphological Examination of Liver and Fat Tissues

The liver and epididymal WAT were removed from the mice, fixed in 10% (*v/v*) paraformaldehyde/phosphate-buffered saline, and embedded in paraffin for H&E staining. The stained areas were viewed under an optical microscope (Nikon, Tokyo, Japan) at 200× magnification.

### 4.7. Statistical Analysis

All data were presented as the mean ± standard error of the mean. Statistical analyses were performed using the statistical package for the social sciences software program SPSS (SPSS Inc., Chicago, IL, USA). Statistically, differences between groups (HFD and ND; and ED and HFD) were determined using Student’s *t*-test.

## 5. Conclusions

In conclusion, our current findings demonstrated that dietary ED attenuated lipogenesis in WAT and increases fecal lipid excretion, thereby reducing adiposity and dyslipidemia. In addition, ED improves NAFLD partly by decreasing hepatic lipogenesis and increasing hepatic FA oxidation. In addition, ED supplementation improves hyperglycemia, hyperinsulinemia, and IR by enhancing glucose utilization and decreasing hepatic gluconeogenesis, as well as improving inflammation. Taken together, these findings indicated that 16 weeks of ED supplementation could be helpful in preventing obesity and metabolic disorders, such as adiposity, dyslipidemia, NAFLD, and IR in HFD-induced obese mice.

## Figures and Tables

**Table 1:** Primer sequences used for RT-qPCR.

Gene	Primer Direction	Primer Sequence
Glyceraldehyde 3-phosphate dehydrogenase (*GAPDH*)	Forward	5′-CAAGTTCAACGGCACAGTCAAGG-3′
	Reverse	5′-ACATACTCAGCACCAGCATCACC-3′
Peroxisome proliferator-activated receptor alpha (*PPARα*)	Forward	5′-CCTGAACATCGAGTGTCGAATAT-3′
	Reverse	5′-GGTCTTCTTCTGAATCTTGCAGCT-3′
PPAR-gamma coactivator 1alpha *(PGC1α)*	Forward	5′-AAGTGTGGAACTCTCTGGAACTG-3′
	Reverse	5′-GGGTTATCTTGGTTGGCTTTATG-3′
*PGC1β*	Forward	5′-GGTCCCTGGCTGACATTCAC-3′
	Reverse	5′-GGCACATCGAGGGCAGAG-3′
Sterol regulatory element-binding transcription factor 1a (*SREBP1a*)	Forward	5′-TAGTCCGAAGCCGGGTGGGCGCCGGCGCCAT-3′
	Reverse	5′-GATGTCGTTCAAAACCGCTGTGTGTCCAGTTC-3′
Acetyl-CoA carboxylase (*ACC*)	Forward	5′- GCCTCTTCCTGACAAACGAG-3′
	Reverse	5′-TGACTGCCGAAACATCTCTG-3′
Fatty acid synthase (*FAS*)	Forward	5′-GCTGCGGAAACTTCAGGAAAT-3′
	Reverse	5′-AGAGACGTGTCACTCCTGGACTT-3′
Steroly-CoA desaturase 1 (*SCD1*)	Forward	5′-CCCCTGCGGATCTTCCTTAT-3′
	Reverse	5′-AGGGTCGGCGTGTGTTTCT-3′
Lipoprotein lipase (*LPL*)	Forward	5′-GACTCGCTCTCAGATGCCCTAC-3′
	Reverse	5′-GCCTGGTTGTGTTGCTTGCC-3′
CD antigen 36 (*CD36*)	Forward	5′-TGGTGGATGGTTTCCTAGCCTTTC-3′
	Reverse	5′-TCGCCAACTCCCAGGTACAATC-3′
Adrenoreceptor beta 3 (*ADRB3*)	Forward	5′-ACCAACGTGTTCGTGACT-3′
	Reverse	5′-ACAGCTAGGTAGCGGTCC-3′
*CPT2*	Forward	5′-GCCTGCTGTTGCGTGACTG-3′
	Reverse	5′-TGGTGGGTACGATGCTGTGC-3′
Cytochrome *c* oxidase subunit 8B (*COX8B*)	Forward	5′-TGTGGGGATCTCAGCCATAGT-3′
	Reverse	5′-AGTGGGCTAAGACCCATCCTG-3′
Uncoupling protein 1 (*UCP1*)	Forward	5′-AGATCTTCTCAGCCGGAGTTT-3′
	Reverse	5′-CTGTACAGTTTCGGCAATCCT-3′
Insulin receptor substrate 2 (*IRS2*)	Forward	5′-CCCATGTCCCGCCGTGAAG-3′
	Reverse	5′-CTCCAGTGCCAAGGTCTGAAGG-3′
Phosphoenol pyruvate carboxykinase (*PEPCK*)	Forward	5′-ATCATCTTTGGTGGCCGTAG-3′
	Reverse	5′-ATCTTGCCCTTGTGTTCTGC-3′
Glucose-6-phosphatase (*G6PC*)	Forward	5′-GGAGGAAGGATGGAGGAAGGAATG-3′
	Reverse	5′-GGTCAGCAATCACAGACACAAGG-3′

## Abbreviations

MDPI	Multidisciplinary digital publishing institute
ACC	Acetyl-CoA carboxylase
AI	Atherogenic index
APO	Apolipoprotein
CPT	Carnitine palmitoyltransferase
DIO	Diet-induced obese
ED	Eriodictyol
FAS	Fatty acid synthase
FER	Food efficiency ratio
FFA	Free fatty acid
GIP	Gastric inhibitory polypeptide
GK	Glucokinase
GLP-1	Glucagon-like peptide-1
G6Pase	Glucose-6-phosphatase
G6PD	Glucose-6-phosphate dehydrogenase
HFD	High-fat diet
HOMA-IR	Homeostasis model assessment of insulin resistance
HTR	HDL-cholesterol-to-TC ratio
H&E	Hematoxylin and eosin
IFN-γ	Interferon-gamma
IL	Interleukin
IPGTT	Intraperitoneal glucose tolerance test
IR	Insulin resistance
IRS2	Insulin receptor substrate 2
ME	Malic enzyme
NAFLD	Non-alcoholic fatty liver disease
NASH	Non-alcoholic steatohepatitis
PAI-1	Plasminogen activator inhibitor-1
PAP	Phosphatidate phosphohydrolase
PEPCK	Phosphoenolpyruvate carboxykinase
PGC1α	PPAR-gamma coactivator 1 alpha
PPARα	Peroxisome proliferator-activated receptor alpha
SREBP	Sterol regulatory element-binding transcription factor
TC	Total-cholesterol
TG	Triglyceride
WAT	White adipose tissue
